# Coordination of quorum sensing with T3SS and T6SS: Bacterial strategies for combating fungal pathogens

**DOI:** 10.1080/21505594.2026.2739060

**Published:** 2026-09-28

**Authors:** Yuanyuan Ma, Anmin Ren, Xiaolei Ji, Yihua Sun, Peng Xue, Liang Yang

**Affiliations:** a Institute for Applied Research in Public Health, School of Public Health, Nantong University, Nantong, China; b School of Medicine, Southern University of Science and Technology, Shenzhen, China; c Nantong Center for Disease Control and Prevention, Nantong, China

**Keywords:** T6SS, T3SS, bacterial-fungal interactions, quorum sensing, antifungal mechanisms

## Abstract

Bacterial-fungal interactions are essential for ecosystem balance, where secretion systems like the Type III Secretion System (T3SS) and Type VI Secretion System (T6SS) play crucial roles. T6SS acts as a multifunctional weapon by delivering antifungal effectors that disrupt fungal cell integrity and metabolism, while T3SS mediates both mutualistic and antagonistic interactions, with mutualistic roles documented primarily in plant-associated bacteria–fungi systems. Quorum sensing (QS) facilitates these interactions by allowing bacteria to adjust their secretion responses based on fungal signals. This review delves into the antifungal mechanisms of T6SS and T3SS and emphasizes QS as a regulatory framework that influences microbial community dynamics. Understanding these relationships can lead to innovative strategies for combating fungal pathogens, contributing to agricultural sustainability and paving the way for future clinical and environmental research.

## Introduction

Bacteria and fungi constitute two of the most abundant and ecologically significant groups within Earth’s microbial communities [1,2]. They are vital in nutrient cycling, promoting soil health, and maintaining host-microbe homeostasis across various organisms [3,4]. These interactions occur in diverse environments such as the gut, rhizosphere, and phyllosphere [5–7]. In their shared habitats, bacteria and fungi form complex networks encompassing a spectrum of relationships, from mutualistic cooperation to aggressive antagonism [8,9]. Fungal hyphae serve as conduits for bacterial dispersal and as substrates for nutrient exchange, providing protection against environmental stresses. Simultaneously, bacterial populations influence fungal physiology through metabolite and effector protein production [9]. Recent advances in microbial ecology and molecular biology have revealed that bacteria–fungi interactions are far more intricate than previously recognized, involving diverse regulatory pathways, specialized secretion machineries, and chemical communication systems. Increasing evidence demonstrates that bacterial antagonistic strategies, particularly secretion system-mediated effector delivery and quorum sensing (QS)-dependent regulation, play essential roles in shaping fungal colonization, competition, and community assembly [10,11]. These findings have significantly expanded our understanding of bacteria–fungi interactions from simple antagonistic or mutualistic relationships toward highly dynamic and evolutionarily driven microbial networks.

Central to these interactions are the secretion systems employed by both bacteria and fungi, which mediate competitive and mutualistic dynamics [12]. These systems enable the transport of toxins, enzymes, and signaling molecules, impacting the extracellular environment and neighboring cells [13]. Importantly, bacteria–fungi interactions rarely occur in isolation in natural ecosystems; instead, they take place within complex polymicrobial communities where multiple bacterial, fungal, and other microbial species coexist and interact simultaneously. In such environments, secretion systems may function beyond pairwise antagonism or cooperation by influencing multiple community members and shaping microbial network structures. In bacteria, type III secretion system (T3SS) and type VI secretion system (T6SS) are crucial for inter-kingdom interactions. T3SS transports effector proteins into the cytoplasm of target eukaryotic cells, affecting fungi and mammals [10,13], while T6SS employs a contractile mechanism to deliver effectors to both prokaryotic and eukaryotic cells [14,15]. Recent research indicates T6SS can target multiple fungal cell components, influencing cell wall, membrane, and metabolic pathways [6,16–18]. Previous reviews have provided valuable summaries of bacteria–fungi interactions, particularly focusing on ecological associations, fungal-associated bacterial communities, or the antimicrobial potential of individual bacterial metabolites and antagonistic mechanisms [19–21].

However, most existing reviews have primarily described interaction patterns or cataloged specific antibacterial and antifungal factors, while relatively little attention has been given to how bacterial secretion systems are integrated with regulatory networks to determine the timing, specificity, and ecological consequences of antifungal activity [22,23]. In particular, the dynamic coordination between secretion systems and bacterial communication mechanisms, such as QS, remains insufficiently explored from an ecological and evolutionary perspective. Although substantial progress has been made in identifying T3SS- and T6SS-dependent antifungal mechanisms, current studies have mainly focused on individual bacterial species or specific effector proteins, leaving the broader ecological significance and evolutionary conservation of these systems insufficiently understood. Moreover, the regulatory mechanisms that determine when and how these secretion systems are activated during bacteria–fungi interactions remain largely unclear. Importantly, secretion systems should not be considered as independent weapons used solely for microbial competition; rather, they represent environmentally responsive regulatory modules embedded within complex bacterial decision-making networks. Their ecological effects are further complicated in polymicrobial environments, where a single secretion system may simultaneously influence multiple microbial competitors, partners, and host-associated organisms, thereby contributing to community-level assembly and stability rather than simple one-to-one interactions. Understanding how bacteria integrate population density signals, environmental cues, and fungal-derived stimuli to regulate effector deployment may provide a more comprehensive explanation of microbial community assembly and stability. As our understanding of these interactions evolves, it’s clear T3SS and T6SS are not isolated systems; they are part of complex networks shaped by evolutionary pressures. QS adds another layer of complexity, allowing bacteria to coordinate activities and adjust phenotypic outcomes through diffusible signaling molecules [11]. QS affects numerous biological processes, including virulence factor expression and influencing bacterial competitiveness against fungi [11]. Furthermore, QS interacts with T3SS and T6SS functions, as signaling molecules modulate their expression and activity in response to environmental cues and population density [6,10]. However, despite increasing evidence linking QS with secretion system regulation, the molecular mechanisms underlying QS-mediated coordination of T3SS/T6SS activation, effector deployment, and ecological outcomes in fungal-associated environments remain poorly characterized. In particular, how these regulatory networks contribute to microbial community stability, fungal pathogen suppression, and host-associated microbial balance represents a critical knowledge gap.

This review adopts a regulatory-network perspective to explore bacterial antifungal strategies, integrating T3SS, T6SS, and QS as interconnected components. We argue that bacterial antifungal activity results from the coordinated regulation of secretion capacity, signaling pathways, and ecological context rather than merely the presence of specific effectors. By examining the antifungal capabilities of T6SS and T3SS within QS-mediated bacterial-fungal interactions, we compare advances and limitations of previous studies, summarize emerging regulatory principles, and highlight unresolved questions regarding evolutionary conservation, ecological functions, and applications. This review emphasizes the functional interconnections among T6SS, T3SS, and QS, proposing that bacterial antifungal strategies are governed by an integrated regulatory network where QS coordinates the activation of secretion systems, effector deployment, and ecological outcomes.

## T6SS: A multifunctional molecular weapon against fungi

The T6SS is one of the most characterized bacterial secretion systems involved in antifungal interactions across diverse bacterial lineages [16,24]. Initial studies demonstrated its antifungal capabilities in *Serratia marcescens*, with T6SS-mediated delivery of two antifungal effectors – Tfe1 and Tfe2 – significantly suppressing fungal growth (Figure 1(A)) [16]. Although both effectors ultimately trigger cell death in fungi, they do so through distinct mechanisms: Tfe1 dissipates plasma membrane potential, whereas Tfe2 disrupts nutrient acquisition and metabolism, inducing autophagy [16]. Proteomic analyses show that translocated Tfe2 leads to metabolic dysfunction in target fungi, characterized by downregulation of amino acid biosynthesis and impaired membrane transport [16]. Various T6SS-delivered antifungal effectors have been identified across bacterial lineages, enhancing therapeutic specificity. For example, TfeC from *Yersinia pseudotuberculosis* degrades chitin in fungal cell walls, directly causing lysis (Figure 1(A)) [6]. This mechanism directly compromises the structural integrity of the fungal cell wall – a characteristic not present in mammalian cells – indicating therapeutic selectivity. Additionally, TseN produced by *Acidovorax citrulli* carries a DNase domain at its C-terminus; upon translocation into the fungal nucleus, this domain degrades chromosomal DNA [18]. TseN exhibits broad-spectrum antifungal activity against various pathogens, including *Candida auris* and *Cryptococcus neoformans*, both of which pose significant clinical challenges due to emerging multidrug resistance [18]. Structural biology investigations have revealed that the DNase domain of TseN defines a novel effector class, whose activity is neutralized by its cognate immunity protein TsiN that binds to and inhibits the DNase domain, preventing self-toxic effects (Figure 1(A)) [18].

**Figure 1.**
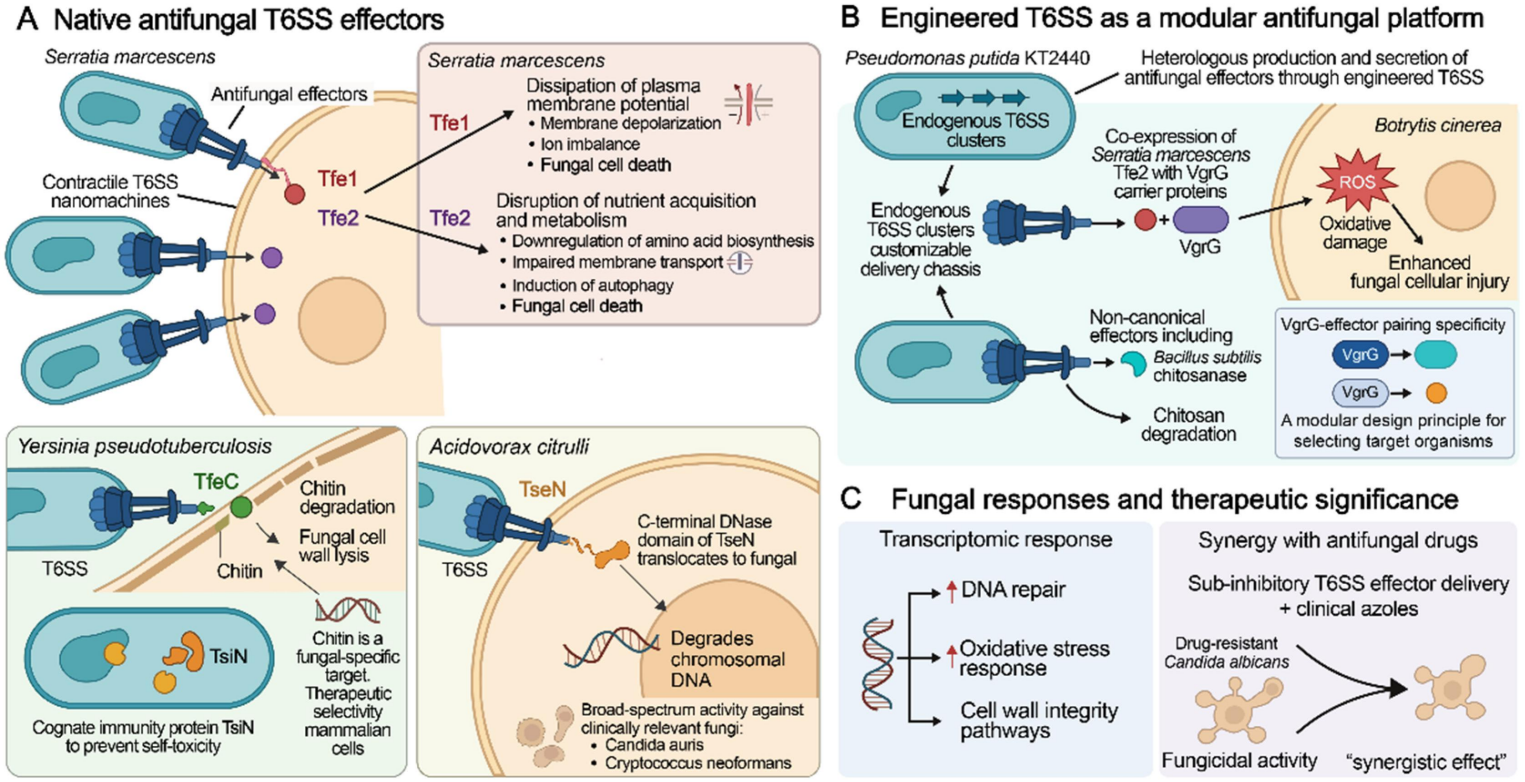
T6SS as a multifunctional molecular weapon against fungi. Gram-negative bacteria use the type VI secretion system (T6SS) to deliver native or engineered antifungal effectors into fungal cells, driving fungal killing and supporting potential therapeutic applications. A. Native antifungal T6SS effectors. *Serratia marcescens* delivers Tfe1 and Tfe2 into fungal cells. Tfe1 disrupts plasma membrane potential and ion homeostasis, whereas Tfe2 impairs nutrient acquisition, metabolism, membrane transport, and autophagy regulation, leading to fungal death. *Yersinia pseudotuberculosis* delivers TfeC to the fungal cell wall, promoting chitin degradation and cell wall lysis. *Acidovorax citrulli* delivers TseN, whose DNase domain enters the fungal nucleus and degrades chromosomal DNA, while TsiN prevents bacterial self-intoxication. B. Engineered T6SS as a modular antifungal platform. Engineered *Pseudomonas putida* KT2440 can serve as a delivery chassis for heterologous antifungal effectors. VgrG-assisted delivery of *S. marcescens* Tfe2 into *Botrytis cinerea* induces ROS accumulation and fungal damage. T6SS can also deliver non-canonical effectors, such as *Bacillus subtilis* chitosanase, to degrade fungal cell wall components, highlighting its modularity and target specificity. C. Fungal responses and therapeutic significance. Fungal cells activate DNA repair, oxidative stress, and cell wall integrity pathways in response to T6SS attack. T6SS-mediated antifungal activity can also synergize with azoles, enhancing killing of drug-resistant *Candida albicans*.

Although Tfe1, Tfe2, TfeC, and TseN are all delivered through the T6SS platform, they represent functionally and structurally distinct effector classes with different cellular targets and ecological implications. Tfe1 provides rapid antifungal activity by disrupting membrane stability, whereas Tfe2 induces progressive physiological stress through interference with metabolic processes. TfeC targets fungal-specific cell wall components, potentially improving target selectivity, while TseN eliminates fungal cells through intracellular DNA damage. Collectively, these differences demonstrate that T6SS antifungal activity is not mediated by a single conserved mechanism but rather by a modular effector repertoire containing diverse catalytic activities, structural features, and target preferences. The ecological range of individual effectors likely depends on both effector properties and accessibility of cellular targets. Effectors targeting conserved processes, such as membrane integrity or DNA stability, may exhibit broader activity, whereas enzymes recognizing specific fungal structures may provide narrower but more selective effects. The presence of immunity proteins, such as TsiN, further highlights the evolutionary balance between effector toxicity and bacterial self-protection. Understanding these structural and functional principles provides a foundation for rational selection and engineering of T6SS effectors for targeted antifungal strategies.

Beyond native systems, the modular architecture of T6SS offers promising engineering opportunities for developing targeted antimicrobial platforms (Figure 1(B)) [25]. For instance, *Pseudomonas putida* KT2440 – a soil bacterium that harbors three endogenous T6SS clusters – has been successfully modified to heterologously produce and secrete antifungal effector proteins [25]. Co-expression of the *Serratia marcescens* Tfe2 effector along with VgrG carrier proteins led to increased levels of reactive oxygen species and enhanced cellular damage in the plant pathogen *Botrytis cinerea* (Figure 1(B)) [25]. Notably, T6SS can be engineered to deliver non-canonical effectors, including proteins not naturally evolved as T6SS substrates [25]. For instance, expressing a chitosanase from *Bacillus subtilis*, an enzyme that degrades fungal cell wall chitosan, in an engineered strain of *P. putida* resulted in T6SS-dependent antifungal activity (Figure 1(B)) [25]. The specificity of effector delivery is determined by VgrG-effector pairing, allowing for modular design in selecting specific VgrG proteins to target particular effectors to intended organisms (Figure 1(B)) [25]. Transcriptomic profiling of fungal cells subjected to T6SS attack has revealed upregulation of genes associated with DNA repair, oxidative stress responses, and cell wall integrity pathways (Figure 1(C)) [18]. Importantly, T6SS-mediated attacks have been shown to act synergistically with azole antifungals, enhancing their efficacy against drug-resistant *C. albicans* (Figure 1(C)) [18]. The combination of sub-inhibitory doses of T6SS effector delivery alongside clinical azoles results in improved fungicidal activity compared to either treatment alone (Figure 1(C)) [18].

However, T6SS-mediated antifungal activity is unlikely to be constitutively active because continuous production of secretion machinery imposes substantial metabolic costs on bacteria. Increasing evidence indicates that T6SS activation is regulated by complex signaling networks involving QS, two-component systems, and environmental sensing pathways [6]. Therefore, T6SS should be viewed not only as a direct antifungal effector delivery system but also as an environmentally responsive output controlled by bacterial regulatory networks. Similar principles may apply to T3SS, suggesting that different secretion systems are coordinated according to ecological conditions rather than functioning as independent antimicrobial mechanisms.

## T3SS: A mediator of mutualistic and antagonistic bacteria–fungi interactions

Following the discussion of T6SS as a direct effector delivery system involved mainly in fungal inhibition, it is important to consider another major secretion system, T3SS, which exhibits broader ecological functions. Unlike T6SS, which primarily mediates contact-dependent antagonistic interactions, T3SS can contribute to both mutualistic adaptation and pathogenic interactions by modulating host or fungal physiology. The functional diversity of T3SS during bacteria–fungi interactions is closely associated with the structural organization of this secretion machinery. Unlike T6SS, which relies on a contractile injection mechanism, T3SS forms a syringe-like nanomachine consisting of a basal body, export apparatus, needle structure, and translocon complex [26,27]. The basal body anchors the secretion apparatus within bacterial membranes, whereas the needle provides a channel for effector transport. Following recognition of host-cell signals, translocon proteins assemble at the target membrane to form a pore, allowing bacterial effectors to access the cytoplasm of eukaryotic cells [26,28]. This highly coordinated architecture determines secretion efficiency, substrate specificity, and host adaptation.

Importantly, structural requirements for T3SS-mediated effector delivery differ among fungal, plant, and animal targets [29,30]. Plant-associated T3SS systems, particularly Hrp1 and Hrp2 families, are adapted for interactions with plant cells and facilitate delivery of effectors involved in host colonization and immune modulation [30–32]. Animal-associated T3SSs generally rely on specialized translocon proteins that directly interact with host plasma membranes to achieve efficient effector injection [33]. However, fungal cells possess rigid cell walls composed of chitin, glucans, and other polysaccharides, which create additional physical barriers compared with animal cells. Therefore, fungal-targeting T3SSs may require additional mechanisms, such as fungal surface recognition, adaptation of secretion or translocation components, and effector activities that facilitate access to fungal cells, although the structural basis of these adaptations remains poorly understood. Comparative structural studies suggest that variations in needle-associated proteins, translocon components, and effector recognition signals contribute to differences in T3SS target specificity [26,27,33]. These structural adaptations may explain why certain T3SSs primarily function in plant-associated interactions, whereas others participate in fungal antagonism or animal pathogenicity. Further structural studies of fungal-associated T3SSs are needed to determine how these secretion systems adapt to interactions with eukaryotic hosts from different kingdoms.

The T3SS plays a crucial role in mediating complex interactions between bacteria and fungi, especially in environments rich in fungal communities, such as the mycosphere and mycorrhizosphere [34]. Current evidence for T3SS-dependent mutualistic interactions with fungi is predominantly derived from plant-associated systems, including mycorrhizal and endophytic contexts. Although T3SS-mediated bacterial–fungal interactions have also been reported in other host-associated environments, clear evidence for T3SS-dependent mutualism with fungi in non-plant environments, such as the mammalian gut, remains limited. Thus, the mutualistic role of T3SS should currently be interpreted primarily within plant-associated ecological contexts rather than as a broadly established mechanism across all bacterial–fungal habitats. Metagenomic studies have shown a higher prevalence of T3SS in fungal-influenced ecosystems compared to conventional bulk soil. This suggests that bacteria equipped with T3SS may have a competitive advantage when coexisting with diverse fungal species [35]. For instance, bacteria like *Pseudomonas fluorescens* utilize their T3SS to interact with ectomycorrhizal fungi, which enhances fungal colonization and positively impacts plant growth (Figure 2(A)) [36]. Additionally, the endosymbiotic bacterium *Candidatus Glomeribacter gigasporarum*, residing within arbuscular mycorrhizal fungi, employs T3SS to modulate the physiology of its fungal host, thereby ensuring a stable endosymbiotic relationship. Bacteria with T3SS capabilities enhance arbuscular endomycorrhization, facilitating nutrient exchange between plants and fungi (Figure 2(A)) [37]. Phylogenetic analyses of T3SS ATPase sequences indicate that the T3SS involved in bacterial-fungal interactions are predominantly affiliated with the Hrp1 and Hrp2 families, which are associated with bacteria found in plant environments [34].

**Figure 2.**
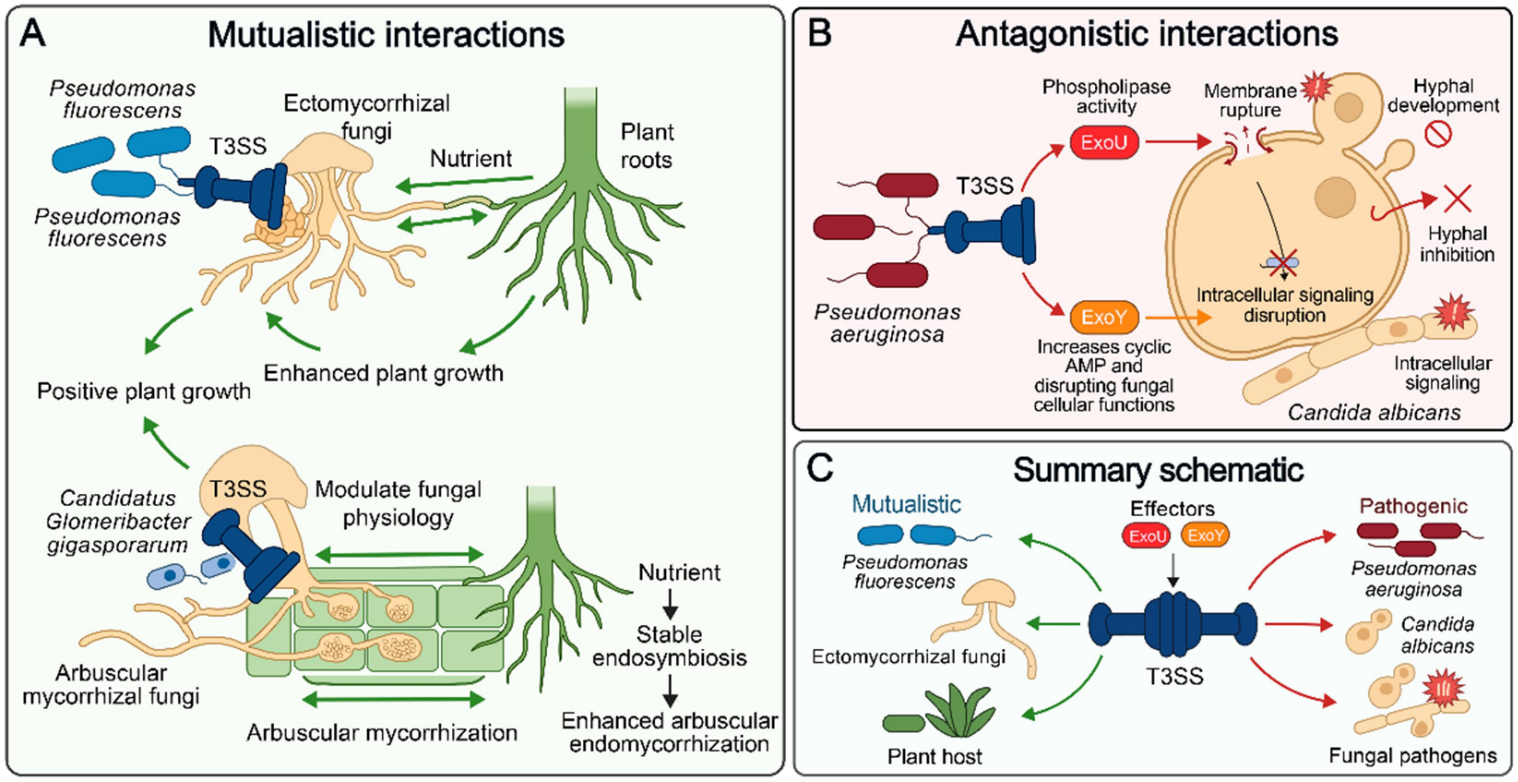
T3SS as a dual-function mediator of mutualism and antagonism in bacterial – fungal interactions. A. Mutualistic interactions. In plant-associated symbiotic systems, bacteria employ T3SS to facilitate fungal colonization, modulate fungal physiology, and promote plant – fungal symbiosis. *Pseudomonas fluorescens* uses T3SS to interact with ectomycorrhizal fungi, thereby supporting fungal growth and contributing to beneficial plant-growth outcomes. *candidatus glomeribacter gigasporarum*, an endosymbiotic bacterium residing within arbuscular mycorrhizal fungi, utilizes T3SS to regulate host fungal functions, maintain stable endosymbiosis, and enhance arbuscular endomycorrhization. B. Antagonistic interactions. In competitive or pathogenic contexts, bacteria deploy T3SS as an offensive apparatus to deliver effector proteins into fungal cells. *Pseudomonas aeruginosa* injects the T3SS effectors ExoU, and ExoY into *Candida albicans*. ExoU exerts phospholipase activity that disrupts the fungal membrane, resulting in cellular leakage and inhibition of hyphal development. ExoY elevates intracellular cyclic amp levels, thereby perturbing fungal cellular functions. C. Conceptual summary. The central T3SS schematic integrates its context-dependent dual functions. On the one hand, T3SS promotes mutualistic bacterial–fungal–plant associations through positive regulatory interactions and nutrient-exchange networks; on the other hand, T3SS mediates antagonistic interactions by enabling bacterial effector delivery into fungal targets.

In contrast to these mutualistic roles, the T3SS also functions as a potent weapon in pathogenic interactions [34]. Recent research has also highlighted the role of T3SS in pathogenic interactions. *Pseudomonas aeruginosa* employs its T3SS to initiate antagonistic interactions with *C. albicans* through two primary effector proteins: ExoU, and ExoY (Figure 2(B)) [10]. ExoU disrupts the fungal membrane via its phospholipase activity, causing cellular leakage and impeding hyphal development. ExoY elevates cyclic AMP levels in fungal cells, disrupting various cellular functions. Collectively, these mechanisms contribute to the antagonistic effects of *P. aeruginosa* on *C. albicans*, showcasing its sophisticated pathogenic strategies (Figure 2(B)) [10]. Overall, the T3SS is essential in bacterial-fungal interactions, with mutualistic functions demonstrated primarily in plant-associated systems, while also facilitating antagonistic interactions with fungal pathogens (Figure 2(C)). This dual function underscores the ecological significance of T3SS-bearing bacteria in shaping fungal dynamics and their overall impact on ecosystem health. Compared with T6SS, which is mainly associated with direct fungal antagonism, T3SS displays greater functional flexibility by mediating both beneficial and detrimental interactions. These differences suggest that bacteria may selectively deploy different secretion systems depending on fungal species, environmental conditions, and ecological objectives. Such selective deployment is likely controlled by upstream regulatory networks, particularly QS-mediated signaling pathways, which coordinate secretion system expression and activity. However, in natural microbial communities, secretion systems may not act exclusively against a single fungal partner. Instead, their activities may contribute to broader polymicrobial interactions by simultaneously influencing multiple microorganisms occupying the same ecological niche.

## QS as an important regulatory layer of T6SS/T3SS antifungal activity

Compared with T6SS, which is mainly associated with direct fungal antagonism, T3SS displays greater functional flexibility by mediating both beneficial and detrimental interactions. These differences suggest that bacteria may selectively deploy different secretion systems depending on fungal species, environmental conditions, and ecological objectives. Such selective deployment is not controlled by QS alone but is determined by integrated regulatory networks involving QS systems, environmental sensing pathways, and secretion-specific transcriptional regulators. QS represents an important regulatory layer that coordinates bacterial behaviors through population-dependent signaling and interacts with other regulatory circuits controlling secretion system expression and activity. QS is a pivotal density-dependent regulatory mechanism that allows microbial communities to synchronize gene expression through the release and detection of signaling molecules [38–41]. In bacteria, QS systems are predominantly characterized by the presence of N-acyl-homoserine lactones (AHLs) in Gram-negative species (Figure 3(A)) and autoinducing peptides (AIPs) in Gram-positive species. These systems are vital for regulating diverse processes, including virulence factor production, motility, biofilm formation, antibiotic synthesis, and bioluminescence [39,42].

**Figure 3.**
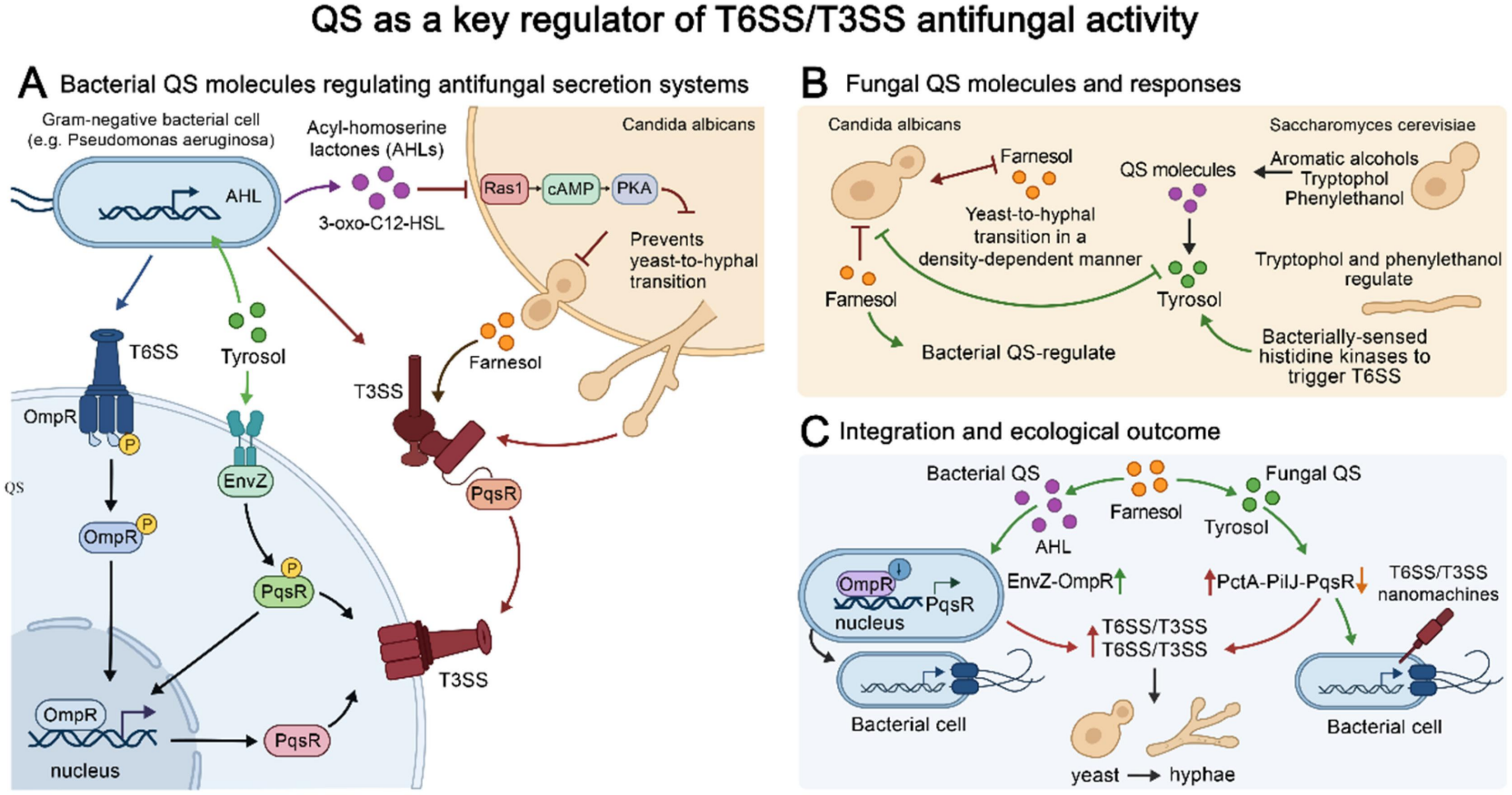
QS-mediated coordination of bacterial antifungal secretion systems and fungal responses. A. Bacterial QS regulation of T6SS/T3SS. *Pseudomonas aeruginosa* produces AHLs such as 3-oxo-C12-HSL, which inhibit the *Candida albicans* Ras1–cAMP–PKA pathway and suppress yeast-to-hyphal transition. Fungal tyrosol activates the EnvZ–OmpR pathway to induce T6SS, whereas farnesol is sensed through the PctA–PilJ–PqsR axis, which integrates chemotactic detection (PctA), surface recognition (PilJ), and QS regulation (PqsR), to promote T3SS-mediated antifungal activity. B. Fungal QS molecules and morphological responses. In *C. albicans*, farnesol inhibits hyphal formation, while tyrosol promotes filamentation. In *Saccharomyces cerevisiae*, aromatic alcohols such as tryptophol and phenylethanol regulate filamentous growth under nitrogen starvation. Fungal QS molecules also modulate bacterial virulence, motility, and biofilm formation. C. Integrated interkingdom outcome. Bidirectional bacterial–fungal QS signaling coordinates T6SS/T3SS activation, fungal morphogenesis, and microbial competition, thereby shaping bacterial–fungal community dynamics.

In fungal interactions, farnesol was the first identified QS molecule produced by *C. albicans*. Farnesol functions as a density-dependent morphogen, inhibiting the transition from yeast to hyphal forms at elevated cell densities (Figure 3(B)) [43]. Subsequent studies identified tyrosol as another QS molecule in *C. albicans*, which promotes filamentation and counteracts the inhibitory effects of farnesol (Figure 3(B)) [44]. Similarly, in *Saccharomyces cerevisiae*, aromatic alcohols such as tryptophol and phenylethanol regulate filamentous growth during nitrogen starvation (Figure 3(B)) [45]. Importantly, the influence of QS on secretion systems is generally mediated through multilayered regulatory networks rather than direct activation of secretion system genes. In many bacteria, QS signals are integrated with dedicated transcriptional regulators that determine secretion system expression, timing, and intensity. For example, in *P. aeruginosa*, T3SS expression is primarily controlled by the AraC-family transcriptional regulator ExsA, which directly activates promoters of T3SS structural and effector genes [46,47]. QS pathways, including the Las, Rhl, and Pqs systems, modulate T3SS activity through regulatory interactions that influence ExsA-dependent transcriptional activation [48]. Thus, QS contributes to T3SS regulation by coordinating bacterial physiological states with secretion-specific regulatory cascades rather than acting as a universal direct switch.

The interplay of QS mechanisms between bacteria and fungi establishes a critical regulatory framework for their communication and behavioral coordination, which ultimately shapes their interactions and enhances adaptability to diverse environments [11]. For example, AHLs produced by *P. aeruginosa*, such as 3-oxo-C12-HSL, interfere with the Ras1-cAMP-PKA signaling pathway in *C. albicans*. This disruption hinders the yeast-to-filamentous form transition, thereby influencing the growth and pathogenicity of *C. albicans* (Figure 3(A)) [49,50]. Conversely, farnesol synthesized by *C. albicans* modifies the QS responses of *P. aeruginosa*, leading to alterations in the production of virulence factors, swarming motility, and biofilm formation (Figure 3(B)) [49,51]. These findings highlight that QS-mediated communication during bacteria–fungi interactions is bidirectional, with microbial signals functioning not only as internal population-density cues but also as interspecies communication molecules that influence competitive and cooperative behaviors.

Two representative examples illustrate how bacterial sensing systems, including QS-associated regulatory pathways, integrate fungal-derived signals to modulate T6SS and T3SS activity (Figure 3(C)). First, the EnvZ histidine kinase in certain bacterial species can sense the fungal signal tyrosol, initiating a phosphorylation cascade that activates the OmpR transcription factor. This results in the upregulation of T6SS, linking fungal signaling to bacterial antifungal responses (Figure 3(C)) [6]. Although this study demonstrates that fungal-derived signals can regulate T6SS activity, the downstream mechanisms connecting OmpR activation with specific T6SS gene clusters may vary among bacterial species and require further investigation. Second, *P. aeruginosa* recognizes farnesol, adjusting its virulence factor expression through a tripartite sensing system (PctA-PilJ-PqsR). This system effectively detects *C. albicans* hyphae and activates an antifungal T3SS, integrating chemotactic detection (PctA), surface recognition (PilJ), and QS regulation (PqsR) to accurately identify fungal morphological forms (Figure 3(C)) [10]. In this process, PqsR functions as one component of a broader regulatory network, while T3SS gene expression ultimately depends on the ExsA-centered transcriptional cascade [10]. Beyond these specific examples, T6SS regulation is also highly context dependent. Different bacterial species employ distinct regulatory architectures involving QS systems, two-component regulatory systems, and stress-response pathways to control T6SS cluster expression [52,53]. Therefore, QS-dependent modulation of T6SS should be viewed as part of a complex regulatory network rather than a conserved direct regulatory mechanism. Collectively, current evidence supports a model in which QS functions as an important regulatory hub within a broader signaling network that integrates environmental and fungal-derived signals to influence multiple secretion systems. Rather than acting as a universal master regulator, QS cooperates with secretion-specific transcription factors and environmental sensing pathways to determine the activation dynamics, specificity, and ecological consequences of T3SS and T6SS responses. In this framework, T6SS and T3SS represent distinct functional outputs of interconnected regulatory networks rather than isolated antifungal mechanisms. This coordinated regulation enables bacteria to balance antagonistic activity, mutualistic interactions, and ecological adaptation, providing a more comprehensive understanding of bacteria–fungi competition and coexistence.

## Conclusions and perspectives

The interactions between bacteria and fungi are fundamental to the ecological balance of diverse habitats, reflecting complex biological relationships that shape microbial community structure and function. Increasing evidence indicates that bacterial secretion systems, particularly T6SS and T3SS, play important roles in determining the outcomes of bacteria–fungi interactions. T6SS represents a versatile antifungal effector delivery platform that enables bacteria to inhibit fungal growth through diverse mechanisms, whereas T3SS displays greater ecological flexibility by mediating both antagonistic and mutualistic interactions depending on bacterial species, fungal partners, and environmental contexts. While T3SS-dependent mutualistic functions have been primarily demonstrated in plant-associated systems, its antagonistic activities against fungal pathogens further highlight the diverse ecological roles of bacterial secretion systems. Importantly, these secretion systems should not be viewed simply as isolated microbial weapons, but rather as components of complex interaction networks that influence microbial community assembly, stability, and adaptation.

Beyond individual secretion systems, regulatory mechanisms determine when and how bacteria deploy these functions in response to environmental conditions. QS functions as an integrative signaling layer that incorporates population density information, environmental signals, and fungal-derived chemical cues into secretion-specific regulatory pathways. Rather than acting as a universal master regulator, QS cooperates with transcriptional regulators, two-component systems, and stress-response pathways to modulate secretion system expression, activation dynamics, and ecological outcomes. The ability of bacteria to respond to fungal signals such as farnesol and tyrosol illustrates the complexity of interkingdom communication and highlights how microbial communities dynamically adjust competitive and cooperative behaviors. Understanding these regulatory networks provides a broader framework for explaining how bacteria balance antagonism, coexistence, and adaptation within fungal-associated environments.

The identification of T6SS antifungal effectors and the discovery of T3SS-mediated beneficial interactions provide promising opportunities for clinical and agricultural applications. T6SS effectors may serve as templates for developing new antifungal strategies, particularly against fungal pathogens exhibiting resistance to conventional antifungal drugs. In addition, engineered bacterial systems with customized effector delivery capabilities may provide potential tools for targeted fungal control, whereas insights into T3SS-mediated beneficial bacteria–fungi interactions may contribute to sustainable agricultural approaches for improving plant health and resilience. However, translating these discoveries into practical applications requires not only technological optimization but also comprehensive evaluation of ecological complexity, biosafety, evolutionary consequences, and long-term stability.

### Critical challenges limiting translational applications

Although T6SS, T3SS, and QS-mediated bacteria–fungi interactions provide valuable opportunities for developing novel antifungal approaches, several challenges remain before these mechanisms can be effectively translated into practical applications. First, secretion systems operate within complex regulatory networks rather than as independent modules [54]. Their expression and activity are controlled by interconnected QS pathways, environmental sensing systems, transcriptional regulators, and stress-response mechanisms. Manipulation of individual regulatory components may therefore cause unintended effects on bacterial fitness, metabolism, or interactions with other microorganisms. For example, regulatory interactions and potential crosstalk between secretion systems indicate that precise control of engineered strains will require a comprehensive understanding of their regulatory architecture. Furthermore, bacterial–fungal interactions rarely occur as isolated pairwise relationships in natural environments. Instead, secretion systems function within polymicrobial communities containing multiple bacterial, fungal, and host-associated organisms. Therefore, future applications must consider how engineered or naturally occurring secretion systems influence broader microbial networks rather than focusing solely on a single target organism. Second, ecological stability and biosafety represent major considerations for the application of antifungal bacteria or engineered microbial systems. Microorganisms showing strong antifungal activity under laboratory conditions may exhibit different behaviors in complex environments due to variations in microbial composition, environmental stress, and host interactions. The introduction of engineered bacteria may alter indigenous microbial communities, affect beneficial microorganisms, or generate unexpected ecological consequences, including potential off-target effects on non-target microbial members [55]. Therefore, successful application requires integration of molecular mechanisms with ecological assessments, including evaluation of environmental persistence, microbial community impacts, and long-term stability. Third, evolutionary adaptation and resistance development remain important limitations. Continuous exposure to bacterial antifungal effectors or QS-based interventions may impose selective pressures that drive fungal populations to evolve resistance mechanisms or alter their ecological strategies. Similar evolutionary processes have been observed in microbial pathogen populations responding to environmental pressures [56]. Therefore, future strategies should incorporate evolutionary monitoring and resistance-risk assessment to ensure long-term effectiveness and sustainability. Beyond these challenges, QS-based approaches face additional limitations related to signal specificity, environmental dependence, delivery efficiency, and regulatory approval. Although QS manipulation provides promising opportunities for controlling microbial behaviors, further studies are required to improve target specificity, stability, and practical applicability [57].

### Priority research directions to address key knowledge gaps

Future research should prioritize several key areas that currently limit both mechanistic understanding and translational applications. First, the identification and characterization of highly selective fungal-targeting T6SS effectors remain an important research priority. Although multiple antifungal effectors have been discovered, their structural determinants, catalytic mechanisms, target specificity, host safety, and ecological impacts remain insufficiently understood. Comparative analyses integrating effector structures, fungal susceptibility mechanisms, and effector–immunity systems will be essential for revealing how molecular architecture determines antifungal activity and ecological function. Beyond T6SS effectors, resolving the structural determinants that regulate T3SS-mediated effector translocation into fungal, plant, and animal cells will provide important insights into the evolutionary adaptation of bacterial secretion systems across different eukaryotic hosts. Second, a deeper understanding of how QS integrates with secretion-specific regulators to control T6SS and T3SS activity is required. Although fungal-derived signals such as farnesol and tyrosol have been shown to influence bacterial behaviors, many regulatory mechanisms have been investigated primarily under simplified laboratory conditions. Future studies should examine QS–secretion system interactions in complex biological settings, including animal infection models, plant–microbe systems, and natural microbial communities. Such studies will clarify whether these regulatory mechanisms contribute primarily to fungal suppression, microbial coexistence, host protection, or context-dependent ecological outcomes. Third, future efforts should address the ecological stability, evolutionary consequences, and translational feasibility of engineered secretion systems. Synthetic biology approaches provide opportunities to design bacteria with targeted antifungal functions; however, predictable performance requires comprehensive evaluation of bacterial fitness, community-level effects, resistance development, and environmental compatibility. In addition, understanding how engineered secretion systems influence surrounding microbial communities will be critical for ensuring ecological safety. Integrating molecular engineering with ecological and evolutionary frameworks will therefore be essential for developing safe and sustainable microbial-based antifungal technologies.

### Integrative perspectives and concluding remarks

In conclusion, bacteria–fungi interactions mediated by secretion systems and regulated by QS represent highly dynamic regulatory networks rather than collections of independent antimicrobial mechanisms. Future progress will depend not only on discovering additional effectors or signaling molecules but also on understanding how secretion systems, regulatory pathways, and microbial communities interact across ecological contexts. Addressing current knowledge gaps in effector specificity, regulatory coordination, ecological validation, and evolutionary stability will determine whether bacteria–fungi interaction-based strategies can be successfully translated into practical applications. By integrating molecular microbiology, microbial ecology, and synthetic biology, future research may enable the rational development of microbial systems capable of controlling fungal pathogens while maintaining ecosystem stability.

## Data Availability

Data sharing is not applicable to this article as no new data were created or analyzed in this study.

## Abbreviations

QS: Quorum sensing
T6SS: Type VI secretion system
T3SS: Type III secretion system
AHLs: N-acyl-homoserine lactones
AIPs: Autoinducing peptides
PQS: *Pseudomonas* quinolone signal
PqsR: *Pseudomonas* quinolone signal receptor
Las: Las quorum sensing system
Rhl: Rhl quorum sensing system
ExsA: Exoenzyme secretion transcriptional activator A
Tfe1: Type VI secretion system antifungal effector 1
Tfe2: Type VI secretion system antifungal effector 2
TfeC: Type VI secretion system effector C
TseN: Type VI secretion system effector N
VgrG: Valine-glycine repeat G protein
TsiN: TseN immunity protein
DNase: Deoxyribonuclease
ROS: Reactive oxygen species
cAMP: Cyclic adenosine monophosphate
PKA: Protein kinase A
EnvZ: Osmolarity sensor histidine kinase EnvZ
OmpR: Osmolarity response regulator OmpR
DNA: Deoxyribonucleic acid
ATPase: Adenosine triphosphatase
*C. albicans*: *Candida albicans*
*C. auris*: *Candida auris*
*C. neoformans*: *Cryptococcus neoformans*
*P. aeruginosa*: *Pseudomonas aeruginosa*
*P. putida*: *Pseudomonas putida*
*P. fluorescens*: *Pseudomonas fluorescens*
*S. marcescens*: *Serratia marcescens*
*B. cinerea*: *Botrytis cinerea*
